# Time to diagnosis for a rare disease: managing medical uncertainty. A qualitative study

**DOI:** 10.1186/s13023-024-03319-2

**Published:** 2024-08-14

**Authors:** Christine Phillips, Anne Parkinson, Tergel Namsrai, Anita Chalmers, Carolyn Dews, Dianne Gregory, Elaine Kelly, Christine Lowe, Jane Desborough

**Affiliations:** 1 School of Medicine and Psychology, Australian National University, 54 Mills Road, Canberra, 2601 ACT Australia; 2 Department of Health Economics, Wellbeing and Society, National Centre for Epidemiology and Population Health, Australian National University, 63 Eggleston Road, Canberra, ACT 2601 Australia; 3 Myositis Association Australia, 14/10 Albany Lane, Berry, NSW 2535 Australia; 4 Immune Deficiencies Foundation Australia, Suite 9, 104 Crown Street, Wollongong, NSW 2500 Australia; 5 Sarcoidosis Australia, Sydney, NSW 2000 Australia

**Keywords:** Rare diseases, Myositis, Primary immune deficiency, Primary immunodeficiencies, Interpretive phenomenological analysis, Qualitative, Diagnosis delay, Uncertainty management theory, Uncertainty

## Abstract

**Background:**

People with a rare disease commonly experience long delays from the onset of symptoms to diagnosis. Rare diseases are challenging to diagnose because they are clinically heterogeneous, and many present with non-specific symptoms common to many diseases. We aimed to explore the experiences of people with myositis, primary immunodeficiency (PID), and sarcoidosis from symptom onset to diagnosis to identify factors that might impact receipt of a timely diagnosis.

**Methods:**

This was a qualitative study using semi-structured interviews. Our approach was informed by Interpretive Phenomenological Analysis (IPA). We applied the lens of uncertainty management theory to tease out how patients experience, assess, manage and cope with puzzling and complex health-related issues while seeking a diagnosis in the cases of rare diseases.

**Results:**

We conducted interviews with 26 people with a rare disease. Ten participants had been diagnosed with a form of myositis, 8 with a primary immunodeficiency, and 8 with sarcoidosis. Time to diagnosis ranged from 6 months to 12 years (myositis), immediate to over 20 years (PID), and 6 months to 15 years (sarcoidosis). We identified four themes that described the experiences of participants with a rare disease as they sought a diagnosis for their condition: (1) normalising and/or misattributing symptoms; (2) particularising by clinicians; (3) asserting patients’ self-knowledge; and (4) working together through the diagnosable moment.

**Conclusions:**

Managing medical uncertainty in the time before diagnosis of a rare disease can be complicated by patients discounting their own symptoms and/or clinicians discounting the scale and impact of those symptoms. Persistence on the part of both clinician and patient is necessary to reach a diagnosis of a rare disease. Strategies such as recognising pattern failure and accommodating self-labelling are key to diagnosis.

## Background

There are estimated to be 7,000 to 8,000 rare diseases [1]. Rare diseases are now identified as an emerging global public health priority affecting 263–446 million people [2]. The majority of rare diseases are genetic in origin and many have an onset in childhood [2]. They may be associated with substantial impairment of physical or mental health, and impact on life expectancy [3].

People with a rare disease commonly experience long delays from onset of symptoms to diagnosis (average time 4–5 years but often much longer) [4]. Rare diseases are challenging to diagnose because they are clinically heterogeneous, and many present with non-specific symptoms common to many diseases. Clinicians may not have encountered patients with the rare condition in their professional practice and may be unfamiliar with the specific condition [1, 5].

The long time to diagnosis often experienced by people with a rare disease is best characterised by uncertainty [6, 7], as they traverse the health system in search of a diagnosis to explain their unresolved symptoms, undergoing multiple clinician visits, investigations, and treatments [3, 7]. People with rare diseases face a significant possibility of misdiagnosis on their journey to diagnosis. In a population-based survey of people with rare diseases in China, adults were more likely to experience misdiagnosis, hence diagnostic delay, than children with rare diseases [8]. Adults who had access to information about rare diseases (for example, through the internet) and those with health insurance were less likely to experience misdiagnosis [8]. Timely diagnosis is important as treatment may be initiated earlier; however, for conditions where treatment may be not be immediately indicated, diagnosis can be helpful in that it resolves uncertainty.

In this paper we focus on the diagnostic journeys undertaken by people with myositis, primary immunodeficiencies (PID) and sarcoidosis. These three conditions were chosen due to their heterogeneous presentation, with known delays in diagnosis. To date, research about diagnostic delay for these diseases has largely drawn on medical record analyses [9–12]. As with most rare diseases, there has been a dearth of qualitative research into the experiences of affected people as they search for a diagnosis. We aimed to explore the experiences of people with myositis, PID, and sarcoidosis from symptom onset to diagnosis to identify factors that might impact receipt of a timely diagnosis.

### Conditions

Idiopathic inflammatory myopathies, commonly referred to as myositis, are a heterogeneous group of rare muscular autoimmune diseases characterised by skeletal muscle inflammation [13]. Muscle weakness is the most common clinical feature, although it can affect other organs including the skin, joints, lungs, heart, and gastrointestinal tract [13]. Prevalence estimates range from 2.9 to 34 per 100,000 [13]. A recent systematic review of the literature found diagnostic delay ranged from 3.48 months to 8 years with a pooled overall mean diagnostic delay of 2.3 years [10].

Primary immunodeficiencies (PID) are a heterogeneous group of rare diseases caused by defects in the immune system [14], attributable to 485 known single gene defects [15]. Using disease registries, the prevalence of PID per 100,000 is estimated to be 4.9 for Australia and New Zealand [16], 9.55 for France [17], 4.43 for Germany [17], 0.54 for the United Kingdom (UK) [17], and 2.3 for Japan [18]. Using population data from insurance claims, the prevalence estimate in the United States (US) was between 41.5 and 50.5 per 100,000 [19]. Time to diagnosis varies. The median delay for common variable immune deficiency (CVID), which has the longest reported diagnostic delay [13], is between 4 and 5 years in many European Union (EU) countries [20], and the UK [21]; however an Australian study [22] found the median delay for CVID was 9 years.

Sarcoidosis is a multisystem granulomatous disease of unknown aetiology which may affect any organ, but predominantly the lungs [23]. Prevalence of sarcoidosis differs greatly across regions from 1 to 5 per 100,000 people in South Korea, Taiwan, and Japan to 60 in the US, and 140–160 in Sweden and Canada [24]. Within the USA, the prevalence is higher among African Americans than other groups [25]. Diagnostic delay has been reported in a recent systematic review of 6 to24 months with a mean delay of 7.93 months [14].

## Methods

### Theoretical framework

We applied the lens of uncertainty management theory (UMT) [26] to tease out how patients experience, assess, manage and cope with puzzling and complex health-related issues while seeking a diagnosis in the cases of rare diseases such as myositis, PID, or sarcoidosis. UMT recognises the emotional load associated with uncertainty. Importantly, the search for knowledge, which is the pre-eminent strategy used to manage medical uncertainty, can result in unsuccessful escalations of exploration and trials of therapy, frustration on the part of patients and clinicians, distrust and despair [27, 28]. While there is an emerging literature on the strategies used by clinicians to manage uncertainty [29, 30], there is a relatively small literature on how people with puzzling symptoms manage uncertainty. Individuals have different levels of acceptance of uncertainty, which may change over time making it important for clinicians and patients to revisit and re-assess patients’ situation and goals [26, 31].

### Study design

This was a qualitative study using semi-structured interviews. Our approach was informed by Interpretive Phenomenological Analysis (IPA) which is well suited to the examination of lived experience of individuals [32, 33] in health settings [34, 35]. It recognises personal experience and its associated meaning as an interpretative exercise that involves both participant and researcher in which the researcher aims to make sense of participants, who are trying to make sense of their world [32, 33].

### Recruitment and participants

We invited three rare disease advocacy groups to join us as research partners: Immune Deficiencies Foundation Australia, Myositis Association Australia, and Sarcoidosis Australia. Each group accepted our invitation and worked with us throughout the study, from framing the research questions to recruiting participants to participating in analysis. The advocacy groups reached out to their members and invited expressions of interest to participate in interviews exploring their personal journeys to diagnosis of their condition. The sampling frame included participants from urban, regional, rural and remote areas of Australia.

### Data collection

Interviews were conducted with 26 participants between November 2021 and April 2022 by TN, AP, or JD. Due to COVID-19 restrictions, two interviews were conducted face to face (attendees wearing masks) and the remaining 24 interviews were conducted by distance. Participants were offered either telephone (*n* = 1) or video call (*n* = 23). Researchers did not experience any differences in quality between interview modes. Five participants chose to attend with a partner, one with a parent, and one participant was the mother of a young child affected by one of the conditions. Participants were asked to describe their symptoms and illness experiences including seeking and receiving a diagnosis and what aspects they found most and least helpful.

### Data analysis

Interviews were audio recorded, professionally transcribed, and anonymised prior to being uploaded to NVivo 12 software [36]. Thematic analysis of the data was conducted using the IPA approach described by Smith and Shinbourne [32], a process that begins as descriptive before moving to a deeper interpretive understanding of the data. Following completion of 10 interviews we conducted a preliminary analysis to determine whether we had reached data saturation and considered whether the interview protocol required refining. Four researchers (JD, CP, AP, TN) familiarised themselves with the transcripts, reading them closely and multiple times. Two researchers (JD, AP) individually coded the same five transcripts drawn from each of the three disease groups and shared their results. Four researchers (JD, CP, AP, TN) then met to discuss and agree on suitable preliminary codes or common themes to apply to the transcripts. A further 16 interviews were conducted until data saturation was reached and no new concepts emerged from the interviews.

Following completion of the interviews, two researchers (JD, AP) conducted an initial deep reading followed by multiple re-readings of the individual transcripts and familiarisation with the data. Additional codes were identified, and some codes merged. All codes were then organised into broad themes reflective of patients’ lived experience, the data reviewed, connections identified, and themes iteratively refined. We identified four themes that described the experiences of participants with a rare disease as they sought a diagnosis for their condition: (1) Normalising and/or misattributing symptoms; (2) Clinician particularising; (3) Asserting patients’ self-knowledge (4) Working together through the diagnosable moment.

Two meetings were held with the whole team to discuss the themes and develop a deeper understanding about participants’ narratives, documenting decision making during this analytical process to enhance reflexivity and ensure rigour.

The Consolidated Criteria for Reporting Qualitative Research (COREQ) guidelines were followed [37]. The study received ethical approval from the Australian National University Human Research Ethics Committee (2021/482).

The research team included three health services researchers who were also clinicians (two GPs [CP, TN], and a registered nurse (JD), and an academic with an extended family member with myositis (AP). All were experienced qualitative researchers. Of the remaining team members, two had a form of myositis and were members of a myositis advocacy group (CL, AC), two had a form of sarcoidosis and were members of a sarcoidosis advocacy group (DG, EK), and one (CD) represented an advocacy group for people with primary immunodeficiencies. Members’ mix of clinical expertise and personal experiences of rare diseases ensured the results were grounded within the data.

### Rigour

We employed Lincoln and Guba’s criteria [38] to establish trustworthiness of the research. Credibility of study findings was established through attaining saturation. Discussions among the team occurred throughout data collection, analysis, and write-up to confirm agreement regarding the approach and findings. Direct quotes from participants sharing a range of viewpoints provided evidence to support our interpretation of the results. Our diverse research team enabled researcher triangulation and authenticity was achieved through verbatim transcription with confirmation of this through listening to recordings. Member checking of interview transcripts and approval of constructed diagnosis timelines was conducted with any changes noted and made as requested. A thick in-depth description of participants’ experiences was provided to aid transferability of our findings and limitations acknowledged.

## Results

There were 26 participants, 31% female. All except one was an adult, with the majority (69%) being over the age of 50 years. 10 participants had been diagnosed with a form of myositis, 8 with a primary immunodeficiency, and 8 with sarcoidosis. Time to diagnosis ranged from immediate to over 20 years (PID), 6 months to 12 years (myositis) and 6 months to 15 years (sarcoidosis). Participants came from all states in Australia and the Australian Capital Territory (ACT). Participant characteristics are presented in Table 1.

**Table 1:** Participant characteristics

ID	Age (years) at interview	Condition	Gender	Location (Australia)	Location classification	Time to diagnosis (years)	Year of diagnosis
001	30–39	Myositis	Female	QLD	Major cities	3–4	2015–19
002	70–79	Myositis	Male	ACT	Major cities	10 plus	2015–19
003	60–69	Sarcoidosis	Female	NSW	Inner regional	10 plus	2015–19
004	60–69	PID	Female	NSW	Major cities	10 plus	2005–09
005	40–49	PID	Female	NSW	Major cities	3–4	2015–19
006	50–59	Sarcoidosis	Female	NSW	Major cities	5–7	2010–14
007	60–69	Myositis	Male	WA	Remote	10 plus	2020–21
008	30–39	Sarcoidosis	Male	VIC	Major cities	5–7	2015–19
009	under 20	PID	Female	QLD	Major cities	less than 0.5	2020–21
010	60–69	PID	Male	VIC	Major cities	10 plus	2010–14
011	60–69	PID	Female	SA	Major cities	10 plus	2005–09
012	50–59	Sarcoidosis	Female	ACT	Major cities	8–10	2005–09
013	20–29	PID	Female	VIC	Inner regional	8–10	2010–14
014	70–79	Myositis	Female	ACT	Major cities	0.5–0.9	2000–04
015	70–79	Myositis	Female	VIC	Outer regional	8–10	2015–19
016	50–59	Sarcoidosis	Female	NSW	Major cities	less than 0.5	2015–19
017	20–29	PID	Male	ACT	Major cities	10 plus	2005–09
018	80 plus	Myositis	Female	NSW	Outer regional	10 plus	2010–14
019	70–79	Myositis	Female	NSW	Major cities	less than 0.5	1995–99
020	70–79	Myositis	Female	NSW	Inner regional	8–10	2015–19
021	70–79	Myositis	Female	NSW	Inner regional	8–10	2005–09
022	70–79	Myositis	Male	WA	Major cities	10 plus	2010–14
023	40–49	Sarcoidosis	Female	VIC	Inner regional	10 plus	2015–19
024	50–59	Sarcoidosis	Male	WA	Major cities	less than 0.5	1995–99
025	50–59	Sarcoidosis	Female	WA	Major cities	1–2	2000–04
026	40–49	PID	Male	ACT	Major cities	0.5–0.9	1995–99

**Note**: PID Primary immune deficiency; ACT Australian Capital Territory, NSW New South Wales, SA South Australia, QLD Queensland, VIC Victoria, WA Western Australia

Participants consulted between 1 and 5 general practitioners (GP) prior to receiving a diagnosis (median, 1 GP). Participants consulted between 1 and 6 specialists (median, 3 specialists) prior to receiving a diagnosis.

Many participants presented with complex, often multisystem, vague, or episodic arrays of symptoms and experienced uncertainty and long delays to diagnosis. Two critical elements that underpinned their experiences were patient self-belief and clinician willingness to reassess symptoms.

### Normalising and/or misattributing symptoms

Almost every participant recounted normalising their symptoms, if they were vague, and particularly if a predominant symptom was fatigue. Those that had rapid diagnoses after onset of disease generally had disabling acute symptoms typical of the disease - for example, sepsis in the case of a child with combined immunodeficiency.

Many participants ascribed their symptoms to over-exertion through working too hard or the stress of parenting and running a busy household, or simply as part of getting older and slowing down. These participants described “carrying on”, pushing through their physical symptoms.

*Yeah*,* so they [two children] were close [born a year apart]*,* and I was working full time*,* and I think I was just sort of… really rundown… Yeah*,* I just sort of continued on.* #004 (PID).

*I worked in childcare in the babies’ room*,* which*,* you know*,* runny noses*,* everything else*,* I thought*,* oh*,* well*,* that’s what it is… I still kept working.* #011 (PID).

Despite the development of significant and limiting symptoms participants described continuing their lives as usual and simply getting on with things.

*I just got used to living with it… I’m getting a bit older*,* getting a bit more tired in my working life… Yeah*,* I just coped with it because I didn’t know any better* #007 (myositis).

Some participants recounted being prompted to act only after their health issues were recognised as abnormal by others, such as family members, friends, or work colleagues.

*So she’d [sister-in-law] seen me in the mornings*,* and she said*,* “Do you know it’s not normal to blow all that?” Like*,* I was so used to it then*,* to blow all that s[tuff] out of your nose every morning… So I came back*,* and I said*,* “Look*,* I’ve got to fix this.” So my GP sent me to an ear*,* nose and throat [specialist].* #011 (PID).

### Clinician particularising: a double discounting

Many participants reported that when they did go to see a clinician, the clinician often focused on the immediate symptom and framed their diagnosis around the proximate organ system, a process we characterised as particularising. Particularising was evident in diagnoses which attributed fatigue to depression, menopause or iron deficiency and respiratory symptoms to asthma or viral infection. Since many participants had managed their uncertainty prior to visiting a clinician by discounting their symptoms, particularising resulted in a “double discounting” of the impact and seriousness of their symptoms. This process of particularising was particularly evident in the case of illness with respiratory presentations.

*I ended up just getting repeated infections… things like tonsillitis*,* pneumonias*,* [I had a] really hard time recovering from those and hitting quite hard and very frequently. So they were just treating each infection as it came up.* #013 (PID).

Recounting their diagnostic journey in retrospect, participants expressed their frustration with what they saw as a failure to engage with other explanations. Many related examples of clinicians locating their diagnosis in one organ system while the patient proposed a multi-system diagnosis. This was reported even in cases where the patient noted a family history of the rare disease.

*[E]very winter I had it and I would go back*,* and I would say*,* “I still have this cough.” They would check my lungs and said*,* “There’s nothing wrong with you.” Sometimes I did have bronchitis… so then they’d put me onto antibiotics. At one time they put me on steroids… it never entered their minds that it could be sarcoidosis… It was the respiratory specialist that I was most disappointed in because I’m going in there every two months saying*,* “My dad has sarcoidosis. I think that’s what’s wrong with me*,*” and he’d say*,* “No*,* it’s not what’s wrong with you. You’ve got reflux.”* #003 (sarcoidosis).

Participants were more likely to cite GPs when discussing particularising, reflecting their frequent contact with GPs often at a time when the condition was less developed. By the time they consulted a specialist, they expected, and often received, a diagnosis of a systemic condition, though not always the correct diagnosis.

*[T]he other neurologist*,* who I felt very comfortable with and everything*,* actually diagnosed me with a completely different illness… so I was a bit surprised*,* but when I went to [name of specialist]… and I told her about it*,* she said*,* oh no*,* you’ve definitely got myositis.* #007 (myositis).

### Asserting patient’s self-knowledge

For most participants, the journey to diagnosis was marked by encounters with clinicians who had arrived at a (mis)diagnosis or had no expectation of a definitive diagnosis. The management of uncertainty often came to involve a contestation with a clinician in which the patient asserted the evidence of their own bodies. In the following account, a participant describes contesting her presumptive diagnosis of asthma.

*And I went to a lung specialist*,* because I had horrendous trouble breathing and everything*,* and he just whacked me with a whole heap of steroids and did a breathing test*,* and it came back normal. And I asked for second and third opinions on this… because I knew something was wrong*,* I knew my body.* #016 (sarcoidosis).

Sometimes the clinician had come to their own accommodation with medical uncertainty while the patient continued to search for more knowledge to reach a definitive diagnosis.

*So*,* after a while*,* I just got fed up… because the doctor after a long while said*,* “Look*,* we don’t really have anything more we can do. So you have interstitial lung disease*,* but we might never know why.” And I just wasn’t happy with that… And I remember asking him… “Could it be anything to do with my immune system because I had these weird things popping up?” and he just laughed it off saying*,* like*,* “Oh*,* no*,* no*,* no*,* no*,* no.”…. So*,* I went back to my GP to get a referral to someone else. #001 (myositis)*.

This account is one of many in which the patient described feeling that their own account was de-legitimised. Participants recounted feeling demoralised, with a number describing they felt as though they were treated as “hypochondriacs”.

*And then all of these doctors that were so-called specialists*,* [in] the lung diseases*,* the way that they treated me and the way that they spoke to me made me feel like I was this whinger person who’s making up all of these things. And I lost trust…. it took two and a half years… of trying to tell other people I needed help*,* that there was something wrong*. #016 (sarcoidosis).

Another participant felt that their pre-existing diagnosed mental health condition seriously impacted their credibility and caused clinicians to focus solely on their mental health and impeded any further investigation:

*I ran out of my painkillers once early because I was in so much pain*,* and my doctor questioned my pain and said*,* “I don’t believe you’re in that much pain*,*” … They didn’t really care because I had anxiety and OCD and that caused them to really overlook me… Getting labelled with anxiety and OCD completely dropped me off the scale of … it was like it made me a non-credible source.* #008 (sarcoidosis).

The resolution of uncertainty in receiving a diagnosis was often accompanied by a sense of relief, attributed by the participants to their trust in their bodily knowledge being affirmed. Discussing their own experience of seeking an explanation for their symptoms, a participant noted

*[I]t really does muck with your head a lot as well. And as crazy as this sounds*,* when you get that diagnosis*,* you’re sort of relieved*,* you’re*,* like*,* I’m not a hypochondriac*,* it’s not in my head.* #006 (sarcoidosis).

Participants recognised that it is not always easy to question the status quo:

*[If I wasn’t] a bit proactive*,* I could have just been lost in the system like a lot of people tend to get that don’t … I mean*,* I worked in hospitals for many years*,* so I know to speak up and question things*,* whereas people that are a bit meek and mild think*,* oh well*,* I’ll just believe the doctor.* #004P (PID).

Just as their first attendances to a clinician had occasionally been triggered by another person de-normalising symptoms, the process of contesting a diagnosis was often supported by an outsider. In the following account, a participant eventually diagnosed with myositis describes nurses in her workplace encouraging her to contest a GP’s working diagnosis.

*I was a volunteer at [name of hospice]*,* and the nurses there said*,* “You need to go to a physician.” So I asked [my GP] about that. “Oh*,* no*,*” he said*,* “they’ll just laugh at you.” So I got nowhere there. And then I used to struggle getting up… I [would] have to use his desk to get up*,* and then one day…I just couldn’t get up. I just struggled and struggled*,* and he said*,* “Oh*,* you’re bad. You are bad.” I said*,* “I’ve been trying to tell you this*,* but you’re not listening.” So he sent me to a Physician at long last.* #020 (myositis).

In this account the GP deflects by stating that the specialist would “laugh at” the referral. Because all referrals to specialists require a GP referral in the Australian health care system, the GP in this account appears to indicate their fear that they, too, would be “laughed at” by the specialist. The patient and the GP are conjoined in the clinician’s concern about their judgement being illegitimate.

### Working together through the diagnosable moment – being in the right place at the right time

The point at which the constellation of symptoms signalled the need for reassessment and possible consideration of a rare disease varied greatly among participants. And, as some reflected, relied on being in the right place at the right time when symptoms of their rare disease were coalescing. One participant with a sick infant was fortunate to be hospitalised at the behest of her GP, where she was attended by a paediatrician who diagnosed the condition.

*She was four months old when she got really sick. And we were just so lucky that we had basically the right people at the right time.* #009 (PID).

A few participants were asymptomatic and diagnosed through serendipity. One was diagnosed with sarcoidosis after it became apparent on an Xray undertaken for a suspected broken rib.

Another was diagnosed with suspected myositis while attending a neurology consultation for her husband.

*I couldn’t stand up when I tried to get out of a chair… Yeah*,* been a regular visitor to the GP*,* and she didn’t know what I had… [W]hen I went up there… the specialist [a neurologist] said*,* “I’m not worried about [husband]*,* he’s got dementia*,* but I’m worried about you.” And I thought to myself*,* what are you worrying about me for? He said*,* “You can’t get out of a chair*,* you can’t stand*,* etc.” and I said*,* ‘No.” And he said*,* “Well*,* I think I know what you’ve got*,*” but he said*,* “We’d better do some tests.”* #021 (myositis).

If clinicians had prior experience of a particular rare disease, this enabled recognition, as was the case of a participant who consulted a locum GP with prior experience of sarcoidosis.

*He’d [GP] seen it before. And it’s only because he’d seen it before and he said… “I think I might know what’s going on.” And that was it.* #023 (sarcoidosis).

Other clinicians were described as setting aside their working diagnosis because they trusted the patients’ representation of their symptoms. These cases represent a different course than that described where participants had to actively assert their bodily knowledge against a clinician’s working diagnosis. One participant characterised this as “being known”. “My GP recognised very early that it was more than arthritis, because [my GP] knew me, and …knew there was something more going on than arthritis” [#014 (myositis)].

In the following account a person finally diagnosed with PID describes having frequent recurrences of chest infections requiring antibiotics, and exacerbating their asthma. In this case, the uncertainty of diagnosis was openly acknowledged by the GP, who “kept testing”. The diagnosable moment came when the participant became seriously ill.

*The doctors… were really starting to get worried at that point*,* because it had moved to*,* like*,* every three months having pneumonia… And that’s when they took some sputum samples and they were actually able to get a sample this time with the illness*,* because sometimes when you cough*,* you don’t always get whatever. And they’d identified Haemophilus influenza*,* which is… a hallmark of people who don’t have immunity to certain kind of things… They also ran bloodwork*,* which included immunoglobulin tests*,* and they noticed that there was some abnormalities there*,* and it was at that point… they were*,* like*,* “You need to see an immunologist.” So that’s when I got referred in.* #005 (PID).

## Discussion

Diagnosis of many rare diseases is complex due to their heterogeneity and number [39, 40]. Rather than focus on building knowledge about specific conditions we posit that it is helpful to consider what may assist more broadly. Our study signals that persistence by both patient and clinician, and re-evaluation of symptoms over time are critical to timely diagnosis. Multiple factors, beyond that of the presenting medical symptoms of the rare condition, can impact receipt of a timely diagnosis.

Our paper studies three diseases whose symptoms may lend themselves to other diagnoses of less serious, episodic conditions, as was experienced by people with PID and sarcoidosis whose initial presentations were of respiratory symptoms. Respiratory illness is the most common acute presentation to general practice in Australia, with acute respiratory illness being managed in 5.1/100 encounters [41]. Australia has the highest reported prevalence of asthma globally, with 11.2% of adults reporting asthma in 2020-21 [42]. Similarly, the fatigue reported in many people’s first presentations with myositis is also a common presentation to general practice, with up to half of family medicine patients in a Canadian study receiving no diagnosis to explain their fatigue [43]. Many of our participants described clinicians using probabilistic reasoning to focus on the most likely cause of their symptoms, which, by definition, was not a rare disease.

This paper draws on the narratives of people who have been diagnosed with a rare disease, and therefore may be seen as being subject to confirmation bias, in that their symptoms are explicable in the light of the final diagnosis. A strength of our study is its temporal dimension; participants describe their long journeys and the gradual move to diagnosis based on a medically coherent summation of symptoms, signs and investigations. Most participants presented with episodic symptoms which could appropriately lend themselves to working diagnoses that were not rare diseases, or to having a cluster of symptoms that were medically undiagnosed. Most people with medically undiagnosed symptoms do not have a known rare disease, and are appropriately managed through collaborative management of the symptoms [44]. The challenge presented by our participants is how to recognise the symptom complexes that are consistent with a rare disease, without involving them in fruitless and expensive investigations and medical opinion-seeking.

In a study of 282 observed consultations by GPs in Germany, researchers outlined the most common strategies used to reach a diagnosis [45]. GPs commonly start with “inductive foraging”, using open-ended questions in which patients state their symptoms and concerns, followed by “triggered routines” asking more specific questions about what they determine to be relevant systems or problems. They may then use probabilistic reasoning (what is the most likely condition), descriptive questioning (asking for more specific details) and deductive testing (asking a question to rule in or rule out a hypothesis). In our study, participants described a few occasions when a clinician had used spot diagnosis – an immediate recognition of the condition – such as the child with PID, and the person whose sarcoid was “recognised” by a locum GP. However, such spot diagnoses, not surprisingly, were infrequent, given the nature of the conditions.

The two strategies most likely to be of relevance to rare diseases are those used the least: pattern failure (recognising where the symptoms, or history did not fit the pattern of a particular disease) and patient self-labelling [45]. Many of our participants described pattern failures. For example, even when probabilistic reasoning could have been used to diagnose a person with sinusitis as having simple sinusitis, its frequent recurrence is an instance of pattern failure, which may alert the clinician to the need to reassess.

Many of our participants also engaged in self-labelling. They raised concerns about a particular disease, perhaps from their own research, or their knowledge of their family history. Self-labelling is clearly an important strategy for managing medical uncertainty; however, it is undermined by the strategy of reassurance frequently used by GPs as part of their consultation. Reassurance was used in almost two thirds of the GP encounters described by Bosner et al. [45] specifically focusing on redirecting patients from concerns about serious diseases. Coia and Morley [46] distinguish affective reassurance from cognitive reassurance. The former is rapid and heuristic, and results in only a transient relief from concern for the patient. Cognitive reassurance may result in a greater reduction in anxiety, and reduced returns by patients for review of symptoms [47].

For patients with rare diseases, affective reassurance may be experienced as glib dismissal of symptoms; cognitive reassurance may be experienced as the clinician’s confirmed belief that their symptoms are undiagnosable. In our study, the period from symptom onset to consulting a doctor was often quite long, as participants wrestled with whether their symptoms were medically meaningful. Being reassured – particularly using affective reassurance – may lead to distrust in the clinician. Both cognitive and affective reassurance may reinforce the patient’s own normalising of symptoms, and delay diagnosis of a rare disease.

We propose that self-labelling and recognising pattern failures are critical to diagnosing rare disease. A patient’s self-labelling may offer a key to understanding the symptoms that are most important to their condition, and allow the clinician to capitalise on the patient’s research [48]. A clinician who conscientiously uses cognitive reassurance should be prepared to re-evaluate the person’s symptoms and their own diagnosis over time. In general, continuity of care between a clinician and patient is useful if the clinician is prepared to engage in re-evaluation of their working diagnosis if the person has indeterminate symptoms but has not been diagnosed and may be consistent with a rare disease. Patients should feel ready to seek reassessment with another doctor, and keep their own records of their results.

### Limitations

Strengths of this study included its focus on the perspectives and experiences of people with myositis, PID, and sarcoidosis from symptom onset to diagnosis of a rare disease, and our coproduction approach that included people with a rare disease as research partners. However, the findings of this study need to be considered within the context of its limitations and it should be noted that only people with a diagnosis of myositis, PID, or sarcoidosis were included and not people with other rare diseases. Care must also be taken with regard to the transferability of the findings due to the sample being drawn from one country and one health system, and the inclusion of only those who spoke English. Further research in this area, including with people for whom English is their second language and with people who have received no diagnosis of their symptoms and continue to live with uncertainty, is needed.

## Conclusion

Our study points to the many challenges of managing medical uncertainty in the period before diagnosis of a rare disease. Journeys to diagnosis frequently involve patients discounting their own symptoms and/or experiencing clinicians discounting their weight and impact. Persistence on the part of both clinician and patient, self-belief by patients, and mutual trust are necessary to reach a diagnosis of a rare disease. Part of this involves using strategies such as recognising pattern failure and acknowledgement of self-labelling.

## Data Availability

The datasets generated and analysed during the current study (deidentified, transcribed interviews) are available from https://datacommons.anu.edu.au/DataCommons/rest/display/anudc:6150?layout=def:display.
